# Changes in lipid levels after 48 weeks of dual versus triple therapy observed in the GARDEL study

**DOI:** 10.7448/IAS.17.4.19554

**Published:** 2014-11-02

**Authors:** Pedro Cahn, Jaime Andrade Villanueva, Jose Arribas, Jose Gatell, Javier Lama, Michel Norton, Patricia Patterson, Juan Sierra Madero, Omar Sued, Maria Ines Figueroa, Maria Jose Rolón

**Affiliations:** 1 Clinical Research, Fundacion Huesped, Ciudad Autónoma de Buenos Aires, Argentina; 2 Unidad de HIV, Hospital Civil de Guadalajara “Fray Antonio Alcalde”, Guadalajara, Mexico; 3 Hospital Universitario La Paz, IdiPAZ, Madrid, Spain; 4 Infectious Unit, Hospital Clínic/IDIBAPS, University of Barcelona, Barcelona, Spain; 5 Clinical Research, Asociación Civil Impacta Salud y Educación (IMPACTA), Lima, Peru; 6 Virology, Global Pharmaceutical Research and Development, AbbVie, Medical Affairs Therapeutic Area, Chicago, IL, USA; 7 Department of Infectious Diseases, Instituto Nacional de Ciencias Médicas y Nutrición Salvador Zubirán, Mexico City, Mexico

## Abstract

**Introduction:**

Treatment with ritonavir-boosted protease inhibitors and nucleoside analogues frequently leads to rises in lipids, which might increase the cardiovascular risk. The aim of this study was to describe changes in lipid levels among HIV positive patients participating in the GARDEL study.

**Materials and Methods:**

The GARDEL study compared the efficacy and safety of a dual therapy (DT) combination of LPV/r 400/100 mg BID+3TC 150 mg BID to a triple therapy (TT) with LPV/r 400/100 mg BID+3TC or FTC and a third investigator-selected NRTI in fixed-dose combination among HIV+ treatment naïve patients. We compared changes in lipid levels from baseline to week 48 in both arms.

**Results:**

Patient's characteristics were well balanced regarding mean baseline total cholesterol (157 mg/dL DT, 154 mg/dL TT), triglycerides (142 mg/dL DT, 139 mg/Dl TT), LDL-C (94 mg/dL DT, 91 mg/dL TT) and HDL-C (36 mg/dL DT, 35 mg/dL TT). Changes in total cholesterol, LDL-C and HDL-C were higher in DT arm, compared to TT (32% DT vs 26% TT for cholesterol; 25% DT vs 16% TT for LDL and 33% DT vs 28% TT for HDL). Increase in triglycerides was higher in TT compared to DT (55% DT vs 92% TT) (Table 1). In TT arm LDL-C and total cholesterol elevations were lower among patients receiving TDF compared to those treated with ZDV or ABC.

**Conclusion:**

Changes in lipid parameters were observed in both arms. Albeit the increase was numerically higher for cholesterol (total and LDL-C) in DT arm while TT arm had higher increases in TG; no difference was observed when week 48 values were compared with the NCEP ATP III goals for cardiovascular risk reduction [1]. So, the DT strategy, even missing the lipid-lowering effect observed with tenofovir, does not seem to add significant risk to patients treated with this novel strategy.

**Table 1:** Lipid Concentrations at Baseline and Week 48 and Mean Percentual Change

	DT (n=214)		
			
	BSL*	W48*	Mean percentage change (%)^a^
Total Cholesterol	157	206	+32
HDL-C	36	48	+33
LDL-C	94	117	+25
Non-LDL-C	120	157	+30
TGs	142	222	+55

* Units:m g/dl, mean

a Percentage increase between baseline and week 48. Mean percentage change was calculated at each specific time point for each individual patient as (concentration [week X] - concentration [baseline]) / (concentration [baseline])×100.
